# Protection from systemic pyruvate at resuscitation in newborn lambs with asphyxial cardiac arrest

**DOI:** 10.14814/phy2.14472

**Published:** 2020-06-29

**Authors:** Vasantha H. S. Kumar, Sylvia Gugino, Lori Nielsen, Praveen Chandrasekharan, Carmon Koenigsknecht, Justin Helman, Satyan Lakshminrusimha

**Affiliations:** ^1^ Department of Pediatrics University at Buffalo Buffalo NY USA; ^2^ Department of Pediatrics University at California Davis Sacramento CA USA

**Keywords:** ATP, cardiac arrest, CMRO_2_, hypoxic‐ischemic injury, newborns, pyruvate, resuscitation

## Abstract

**Background:**

Infants with hypoxic‐ischemic injury often require cardiopulmonary resuscitation. Mitochondrial failure to generate adenosine triphosphate (ATP) during hypoxic‐ischemic reperfusion injury contributes to cellular damage. Current postnatal strategies to improve outcome in hypoxic‐ischemic injury need sophisticated equipment to perform servo‐controlled cooling. Administration of intravenous pyruvate, an antioxidant with favorable effects on mitochondrial bioenergetics, is a simple intervention that can have a global impact. We hypothesize that the administration of pyruvate following the return of spontaneous circulation (ROSC) would improve cardiac function, systemic hemodynamics, and oxygen utilization in the brain in newborn lambs with cardiac arrest (CA).

**Methods:**

Term lambs were instrumented, delivered by C‐section and asphyxia induced by umbilical cord occlusion along with clamping of the endotracheal tube until asystole; Lambs resuscitated following 5 min of CA; upon ROSC, lambs were randomized to receive pyruvate or saline infusion over 90 min and ventilated for 150 min postinfusion. Pulmonary and systemic hemodynamics and arterial gases monitored. We measured plasma pyruvate, tissue lactate, and ATP levels (heart and brain) in both groups.

**Results:**

Time to ROSC was not different between the two groups. Systolic and diastolic blood pressures, stroke volume, arterial oxygen content, and cerebral oxygen delivery were similar between the two groups. The cerebral metabolic rate of oxygen was higher following pyruvate infusion; higher oxygen consumption in the brain was associated with lower plasma levels but higher brain ATP levels compared to the saline group.

**Conclusions:**

Pyruvate promotes energy generation accompanied by efficient oxygen utilization in the brain and may facilitate additional neuroprotection in the presence of hypoxic‐ischemic injury.

## Graphical abstract

Current strategies to improve outcome in birth asphyxia require sophisticated servo‐controlled cooling equipment. Administration of intravenous pyruvate, an antioxidant with favorable effects on mitochondrial bioenergetics is a simple intervention that may have a protective effect in asphyxial cardiac arrest.

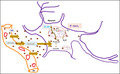

## 1 INTRODUCTION

Infants with perinatal asphyxia often require cardiopulmonary resuscitation (CPR). Infants administered delivery room CPR (DR‐CPR), defined as chest compressions or epinephrine, have worse outcomes in terms of death or neurological impairment (Handley, Sun, Wyckoff, & Lee, 2014; Kanik et al., 2009). Neonatal cardiac arrest (CA) imposes severe, global ischemia predominantly on the heart and the brain contributing to higher mortality and morbidity (Martin‐Ancel et al., 1995). Of particular concern in the early recovery period is the postresuscitation syndrome (Cerchiari, Safar, Klein, Cantadore, & Pinsky, 1993), wherein the injured heart fails to adequately perfuse its tissue or that of the peripheral organs such as the brain (Behringer et al., 2002), leading to multiorgan failure. Cardiovascular dysfunction occurs in 29%–67% of asphyxiated neonates, often requiring inotropic support with poor outcomes (Martin‐Ancel et al., 1995; Shah, Riphagen, Beyene, & Perlman, 2004).

Oxidative stress plays a crucial role in the evolution of hypoxia‐ischemia (HI). Oxidative damage to mitochondria, a principal source and target of reactive oxygen species (ROS), results in damage to mitochondrial membranes, enzymes, and respiratory chain components culminating in the impairment of adenosine triphosphate (ATP) production. Moreover, ROS interacts with physiologic signaling mechanisms and can initiate apoptotic or necrotic cell death during reperfusion, contributing to cellular damage (Ten & Starkov, 2012). Myocardial phosphorylation potential collapses and glutathione (GSH) redox state decreases sharply by 5 min into CA, initiated by severe depletion of energy reserves (Sharma et al., 2005).

Pyruvate, a natural metabolic fuel, is a free radical scavenger that is at the crossroads of crucial metabolic pathways—energy production and scavenging ROS. Exogenous pyruvate by increasing cytosolic ATP phosphorylation potential enhances the myocardial inotropic state (Mallet, Sun, Knott, Sharma, & Olivencia‐Yurvati, 2005). The α‐keto‐carboxylate structure of pyruvate enables it to neutralize peroxides and peroxynitrite (Crestanello, Kamelgard, & Whitman, 1995; Vasquez‐Vivar, Denicola, Radi, & Augusto, 1997). Pyruvate attenuates H_2_O_2_‐induced ROS formation and cell death in a dose‐dependent manner (Wang et al., 2007). Exogenous pyruvate significantly increases endogenous respiration as a consequence of oxidative metabolism (Schuh et al., 2011), suppresses superoxide production by submitochondrial particles, and attenuates oxidative stress‐induced collapse of the mitochondrial membrane potential (Wang et al., 2007). Mitochondrial metabolism contributes to pyruvate's antioxidant character by increasing GSH (Mallet et al., 2005), hence stabilizing the inner mitochondrial membrane.

The metabolic improvements contributed by pyruvate support the postarrest recovery of cardiac electromechanical performance leading to improvements in systemic perfusion and oxygenation. Pyruvate by resuscitating the myocardial physiology at the cellular level may be critical for a successful CPR, especially in the presence of asphyxia. Pyruvate alters the fundamental bioenergetics of mitochondria in the early phase of HI reoxygenation, potentially contributing to cardioprotective and neuroprotective effects. We hypothesize that stabilization of the myocardium by pyruvate following the return of spontaneous circulation (ROSC) in newborn lambs with asphyxial CA may have a beneficial impact on the systemic and pulmonary hemodynamics, contributing to improvements in tissue oxygenation of the vital organs of the body.

## 2 METHODS

### 2.1 Animal preparation

The University at Buffalo's Institutional Animal Care and Use Committee approved the asphyxial CA model in newborn lambs. This model of asphyxial arrest by umbilical cord occlusion in newborn lambs is extensively studied and described previously (Vali et al., 2017a, 2017b). Briefly, time‐dated term pregnant ewes (139‐ to 141‐day gestation; full term ~147 days) purchased from May Family Enterprises and fasted overnight. Anesthesia induced with intravenous diazepam and ketamine; ewes intubated and ventilated with 21% O_2_ and 2%–3% isoflurane and continuously monitored with a pulse oximeter and end‐tidal CO_2_ monitor. Following the cesarean section, fetal lambs were partially exteriorized, intubated, and excess fetal lung fluid drained by gravity. Endotracheal tube (ETT) was occluded to prevent gas exchange during the asphyxial period. Catheters inserted into the jugular vein (for fluid resuscitation and medication administration) and right carotid artery (blood sampling). A 2‐mm flow probe (Transonic Systems Inc) placed around the left carotid artery for measurement of carotid blood flow. A left thoracotomy performed and a 4‐mm probe placed around the left pulmonary artery for measurement of pulmonary blood flow. All hemodynamic variables and EKG monitoring were assessed by ECG100C (Biopac Systems, Inc) with Acknowledge Software for the estimation of hemodynamic variables. The lambs placed on radiant warmer after clamping of the umbilical cord. An umbilical arterial catheter was inserted to monitor blood pressures continuously.

### 2.2 Experimental protocol

Five minutes of asystole was observed before initiating resuscitation. We defined asystole as the absence of carotid blood flow, arterial blood pressure, and heart rate. Resuscitation began with the removal of the ETT occluder followed by ventilation with 21% O_2_ using a T‐piece resuscitator. After 30 s of uninterrupted ventilation, chest compression performed at a ratio of 3:1 (three compressions to one ventilation breath) for 120 events/min. Inspired oxygen was increased to 100% upon initiation of chest compressions. We defined successful ROSC as a heart rate of >100/min and mean blood pressure of >30 mmHg. Epinephrine administered every 3 min as per Neonatal Resuscitation Program guidelines until ROSC achieved. We discontinued all resuscitative efforts for failure to achieve ROSC after 10 min of commencement of CPR. Lambs with successful ROSC were placed on mechanical ventilation; PIP and rate were adjusted based on tidal volumes (goal: 8–9 ml/kg) and PaCO_2_ (goal 40–50 mmHg). FiO_2_ was adjusted to maintain preductal saturations of 85%–95%. Following ROSC, and a brief period of stabilization, the lambs were randomized into two groups using opaque sealed envelopes: the pyruvate group or the placebo group and monitored for 4 hr with q 15‐min arterial blood gases. The laboratory personnel was not aware of the treatment group, as blinding of the treatment groups performed.

### 2.3 Pyruvate administration

Lambs randomized to pyruvate group received sodium pyruvate (SP) (loading dose: 100 mg/kg; infusion: 10 mg kg^−1^ min^−1^ for 90 min) via the jugular line based on previous studies (Kristo et al., 2004). Fresh pyruvate solution was prepared immediately before the administration of the solution on the day of the experiment. The solution was protected from light by an aluminum wrap during infusion. Lambs randomized to the placebo group received an equal volume of normal saline over 90 min. The lambs were monitored for 2½ hr after infusion with pyruvate or saline. The lambs were euthanized by pentobarbital administration (100 mg/kg) (Fatal‐Plus; Vortech Pharmaceuticals) at the end of the experiment.

### 2.4 Physiologic measurements

The physiologic parameters recorded included heart rate, systemic blood pressure, left pulmonary arterial blood flow, left carotid blood flow, jugular venous, and preductal (right carotid) arterial blood gases, and oxygen saturation. Laboratory parameters assessed included hemoglobin, electrolytes, glucose, and lactate in the blood. We calculated the following physiologic indices using the above measurements.

### 2.5 Stroke volume

Cardiac output (CO) calculated using left pulmonary arterial (LPA) flow. Right pulmonary arterial (RPA) flow was estimated to be 1.5‐fold LPA flow.

CO (ml kg^−1^ min^−1^) = LPA flow + RPA flow (1.5 × LPA flow)/body weight.

Stroke volume was deduced from the cardiac output (CO/HR, expressed as ml kg^−1^ beat^−1^).

Arterial Oxygen Content (CaO_2_) (ml/dl) = (Hb × 1.36) (SaO_2_)/100 + (PaO_2_ × 0.0031); Hb = hemoglobin (g/dl); SaO_2_ = arterial oxygen saturations (%); PaO_2_ = arterial oxygen tension (mmHg).

Venous Oxygen Content (CvO_2_) (ml/dl) = (Hb × 1.36) (SvO_2_)/100 + (PvO_2_ × 0.0031); Hb = hemoglobin (g/dl); SvO_2_ = jugular venous oxygen saturations (%); PvO_2_ = venous oxygen tension (mmHg).

Cerebral Oxygen Delivery (CDO_2_) (ml kg^−1^ min^−1^) = Carotid blood flow (CBF) × CaO_2_.

Cerebral Metabolic Rate of Oxygen (CMRO_2_) (ml kg^−1^ min^−1^) = (CaO_2_ − CvO_2_) × CBF.

### 2.6 ATP assay

Whole blood (0, 30, 90, and 180 min), myocardial, and brain (frontal area gray matter of left hemisphere) samples collected for ATP assay. Samples were flash‐frozen in liquid nitrogen immediately upon dissection or collection. Both whole‐blood and tissue samples were homogenized/deproteinated using 10% trichloroacetic acid and extracted with ether. The extracted samples were then analyzed using ATP/adenosine diphosphate (ADP)/adenosine monophosphate (AMP) Assay kit (Biomedical Research Service) per manufacturer's protocol and analyzed using a BioTek Synergy HT plate reader. The assay uses the ATP dependence of the light‐emitting, luciferase‐catalyzed oxidation of luciferin for the measurement of extremely low concentrations of ATP. Results normalized to starting weight (pmols/mg tissue) and starting volume (pmols/µl of whole blood).

### 2.7 Lactate and pyruvate measurements

Frozen samples of plasma from newborn asphyxiated lambs at the ROSC (0 min, baseline); end of pyruvate infusion (90 min) and 1 hr after pyruvate infusion (150 min) were used to estimate pyruvate by a fluorescent‐based pyruvate assay (No.700470, Cayman Chemicals) and expressed as nmol/ml plasma. Lactate measurements were performed by a colorimetric assay (Biomedical Research Service) in frozen heart and brain tissue and expressed as ng/mg tissue.

### 2.8 Statistical analysis

Unpaired Student's *t* test analyzed data for two‐group comparisons or by ANOVA with Fisher's post hoc analysis for more than two‐group comparisons using Statview software (Abacus Concepts). Changes over time between the two groups analyzed by ANOVA repeated measures. Values expressed as mean ± *SEM* with *n* representing the number of animals studied. Significance accepted at *p* < .05.

## 3 RESULTS

We had seven lambs in the control group and eight lambs in the pyruvate group following asphyxial CA with ROSC (Table 1). There were no differences in birth weight, gender distribution, the severity of acidosis at arrest, and time to ROSC between the two groups (Table 1). Arterial blood gases after ROSC, at the beginning of pyruvate infusion (0 min), end of pyruvate infusion (90 min), and in the postpyruvate monitoring period (120, 180, and 240 min) are shown in Table 1. There were no differences in blood gas parameters such as PaCO_2_, PaO_2,_ and base deficit between the two groups during the postresuscitation period.

**TABLE 1:** Blood gases after administration of intravenous sodium pyruvate in newborn lambs with asphyxiated cardia arrest

Parameter	Saline group (*n* = 7)	Pyruvate group (*n* = 8)
Birth weight (g)	4,193 ± 832	3,579 ± 408
Males (%)	3 (42.8%)	4 (50%)
ROSC (s)	355 ± 86	272 ± 159

Time of ABG	pH	PaCO_2_	PaO_2_	Base deficit	pH	PaCO_2_	PaO_2_	Base deficit
Arrest gas	6.85 ± 0.06	132 ± 21	8 ± 4	16.3 ± 1.8	6.85 ± 0.05	133 ± 12	7 ± 6	15.2 ± 3.9
@ ROSC	6.95 ± 0.07	78 ± 24	58 ± 34	16.7 ± 3.2	6.92 ± 0.08	86 ± 29	89 ± 77	17.8 ± 3.9
0 min	7.24 ± 0.05	34 ± 7	93 ± 46	11.8 ± 1.8	7.27 ± 0.11	33 ± 9	77 ± 25	10.4 ± 4.1
90 min	7.29 ± 0.08	33 ± 9	82 ± 37	9.8 ± 1.7	7.29 ± 0.08	30 ± 7	75 ± 30	10.3 ± 4.3
120 min	7.32 ± 0.07	29 ± 6	75 ± 38	9.4 ± 1.8	7.26 ± 0.07	32 ± 6	65 ± 23	10.7 ± 4.4
180 min	7.31 ± 0.08	30 ± 5	85 ± 36	10.1 ± 2.5	7.27 ± 0.13	30 ± 7	90 ± 77	10.1 ± 5.8
240 min	7.32 ± 0.09	30 ± 6	73 ± 26	9.7 ± 3.1	7.27 ± 0.16	30 ± 5	68 ± 21	11.2 ± 6.4

Note

Values expressed as *M* ± *SDM*. Pyruvate infusion from 0 to 90 min. Postpyruvate observation: 90–240 min.

Abbreviations: @ ROSC, gas soon after return of spontaneous circulation; ABG, arterial blood gas; Arrest gas, after asphyxial cardiac arrest; ROSC, return of spontaneous circulation.

### 3.1 Hemodynamics

**FIGURE 1:**
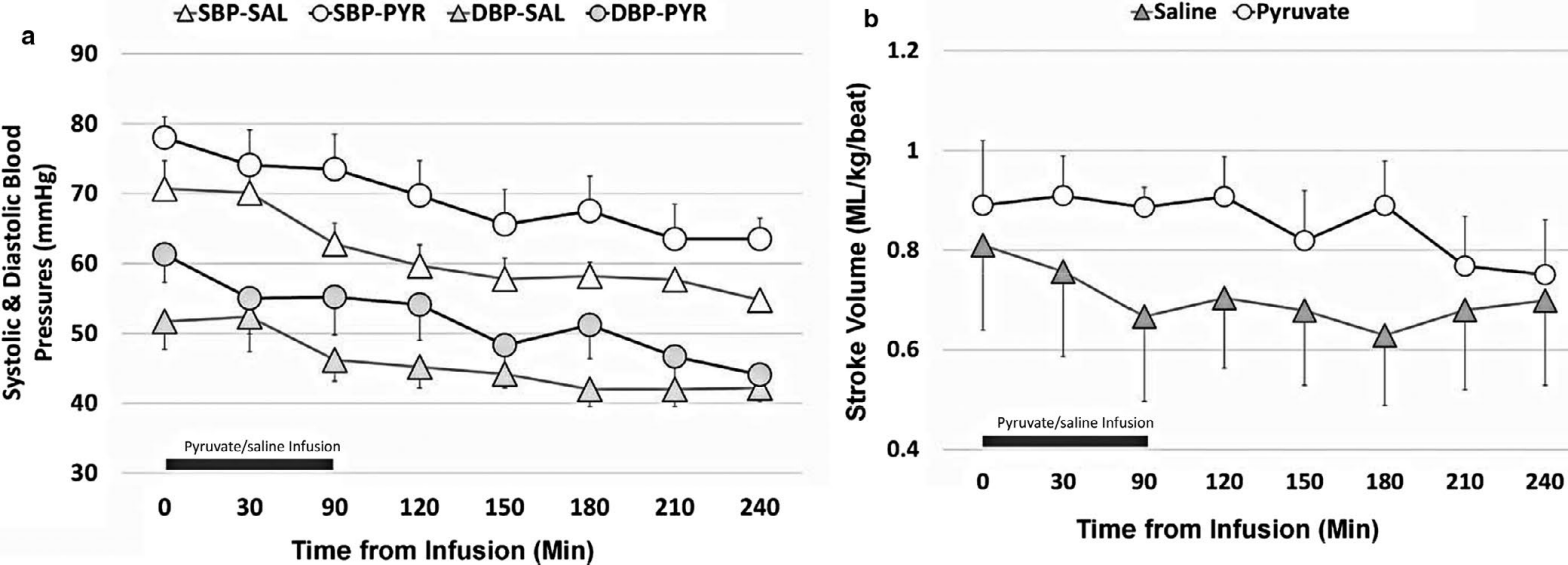
Systemic blood pressure (a) and stroke volume (b) in newborn lambs with asphyxial cardiac arrest. Systolic (open marker) and diastolic (shaded marker) blood pressures were not different in lambs administered pyruvate (circles) or saline (triangles). Similarly, stroke volume was not different between the two infusion groups (pyruvate: *open circles*; saline: *shaded triangles*). The bar above the *X*‐axis represents the infusion of pyruvate or saline (90 min). Values expressed as *M* ± *SEM* (saline: *n* = 7; pyruvate: *n* = 8)

**FIGURE 2:**
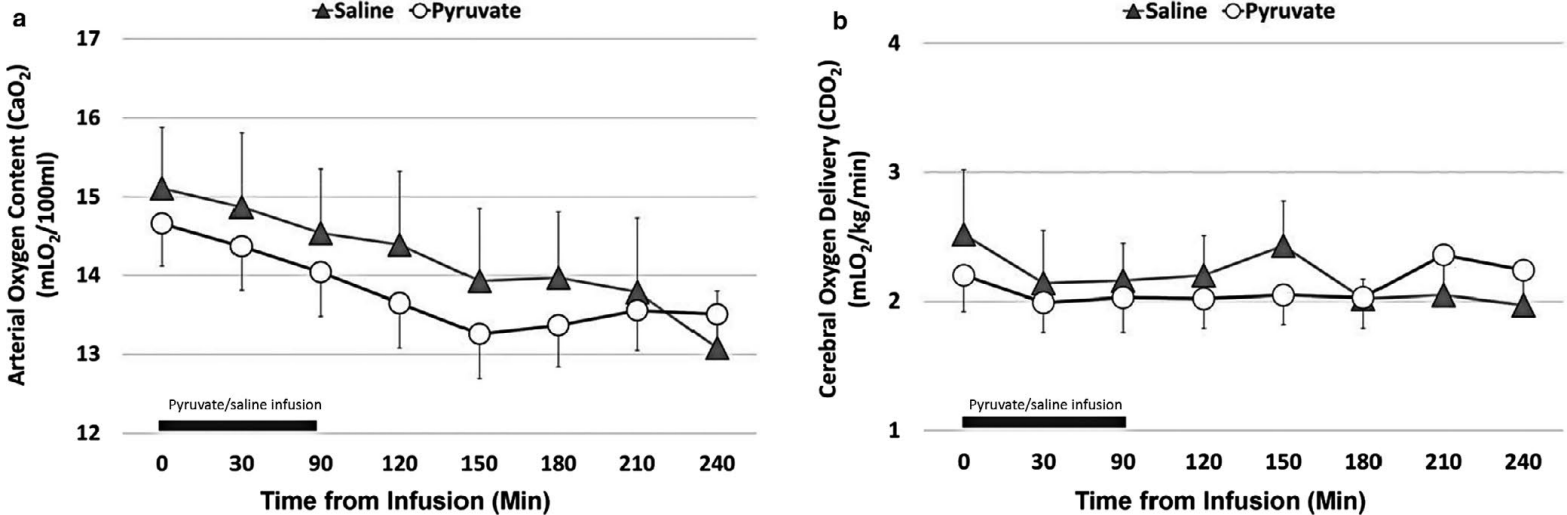
Arterial oxygen content (CaO_2_) (a) and cerebral oxygen delivery (CDO_2_) (b) in newborn lambs with asphyxial cardiac arrest. Both arterial oxygen content and cerebral oxygen delivery were not different between the two infusion groups (pyruvate: *open circles*; saline: *shaded triangles*). The bar above the *X*‐axis represents the infusion of pyruvate or saline (90 min). Values expressed as *M* ± *SEM* (saline: *n* = 7; pyruvate: *n* = 8)

**FIGURE 3:**
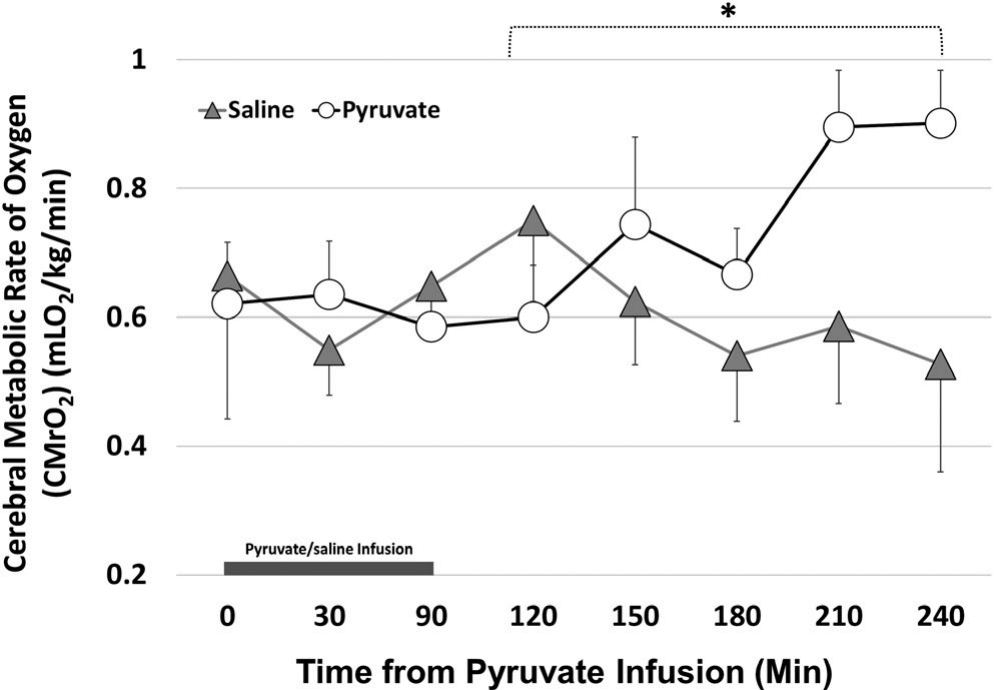
The cerebral metabolic rate of oxygen (CMRO_2_) in newborn lambs with asphyxial cardiac arrest. The cerebral metabolic rate of oxygen was significantly higher postpyruvate infusion compared to the saline administered group (*p < .05 vs. saline group; repeated measures ANOVA). (pyruvate: *open circles*; saline: *shaded triangles*; values expressed as *M* ± *SEM*; saline: *n* = 7; pyruvate: *n* = 8)

Systolic and diastolic blood pressures were not different between the two groups over time (Figure 1a). Stroke volume, as calculated, was not different between the two groups over time (Figure 1b). Similarly, arterial oxygen content (CaO_2_) (Figure 2a) and cerebral oxygen delivery (CDO_2_) (Figure 2b) were not different between the two groups over time. The cerebral metabolic rate of oxygen (CMRO_2_) was significantly higher following pyruvate infusion compared to the control group (Figure 3, *p* < .05 vs. control group, unpaired *t* test).

### 3.2 Lactate and pyruvate measurements

Tissue lactate in the heart and brain tissue was not different between the saline and the pyruvate groups (Table 2). Plasma pyruvate was not different at baseline (0 min) and the end of pyruvate infusion (90 min) between the two groups (Table 2). However, plasma pyruvate was significantly lower in the pyruvate group compared to the saline group an hour after the infusion of pyruvate (*p < .05 vs. saline group, unpaired *t* test; Table 2).

**TABLE 2:** Lactate and pyruvate measurements in tissues and plasma, respectively, following infusion of pyruvate in a model of perinatal asphyxia in newborn lambs

Measurement	Saline group (*n* = 6)	Pyruvate group (*n* = 6)
Tissue lactate (ng/mg tissue)		
Heart	35.7 ± 10.2	30.7 ± 9.6
Brain	29.8 ± 4.2	27.2 ± 5.8
Plasma pyruvate (nMols/ml)		
0 min (at ROSC)	75.8 ± 9.1	76.1 ± 17.7
90 min (end of pyruvate infusion)	88.8 ± 33.0	79.4 ± 19.5
150 min (60 min postpyruvate Infusion)	134.6 ± 50.5	75.6 ± 15.0*

Note

Values are *M* ± *SDM* and expressed as ng/mg tissue (tissue lactate) and in nMols/ml (plasma pyruvate). Pyruvate infusion started at 0 min of age (baseline) for 90 min.

Abbreviation: ROSC, return of spontaneous circulation.

* *p* < .05 versus Saline group, unpaired *t* test.

### 3.3 ATP measurements

**FIGURE 4:**
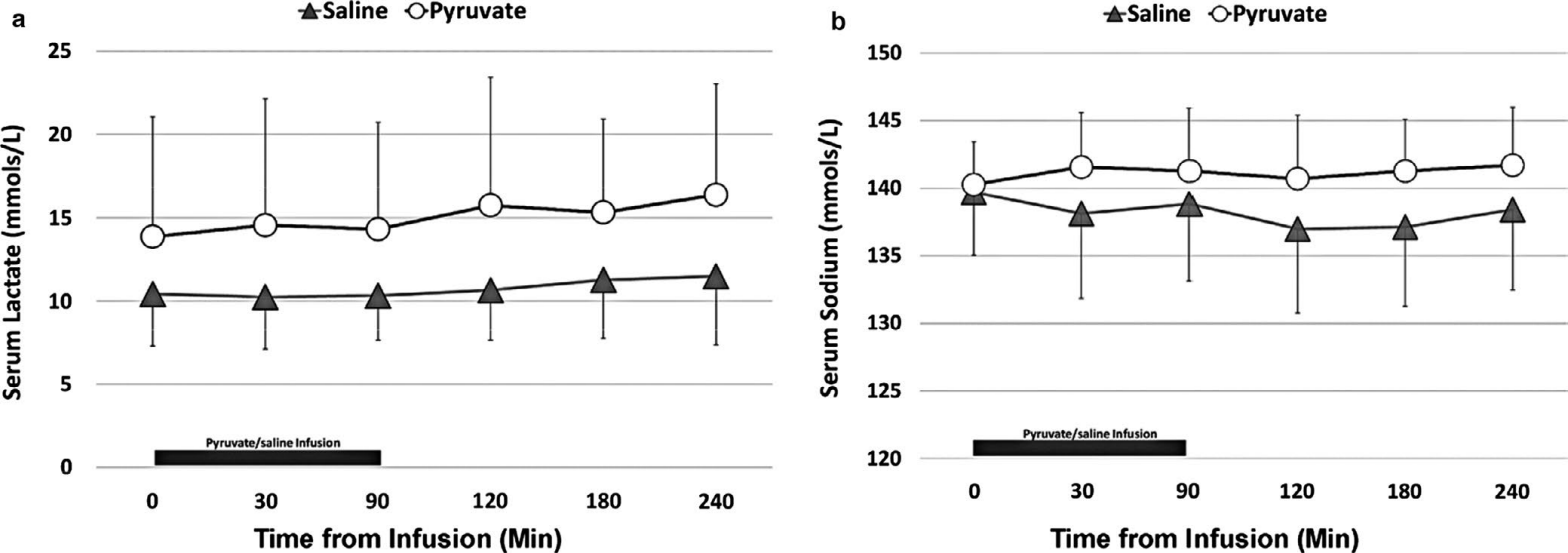
Serum lactate (a) and serum sodium levels (b) in newborn lambs with asphyxial cardiac arrest. Serum lactate and serum sodium levels were not different between the pyruvate and the control groups (pyruvate: *open circles*; saline: *shaded triangles*; values expressed as *M* ± *SD*; saline: *n* = 7; pyruvate: *n* = 8)

Blood ATP measurements at baseline (0 min), during (30 and 90 min), and after pyruvate infusion (180 min) were not different between the pyruvate and the saline groups (Table 3). Similarly, myocardial ATP concentrations were not different between the two groups at the sacrifice. However, brain ATP concentrations were significantly higher in the pyruvate group compared to the control group (Table 3; *p < .05 vs. control group, unpaired *t* test). Serum sodium and lactate were not statistically different between the two groups (Figure 4).

**TABLE 3:** Changes in ATP, ADP, and AMP levels in blood, myocardial, and brain tissue following pyruvate infusion in a model of perinatal asphyxia in newborn lambs

Tissue	Saline group (*n* = 6)	Pyruvate group (*n* = 6)
Blood ATP measurements (pmols/µl)		
0 min (baseline)	2.84 ± 0.46	3.01 ± 0.31
30 min	2.93 ± 0.56	2.84 ± 0.14
90 min	2.98 ± 0.50	3.20 ± 0.42
180 min (postpyruvate infusion)	2.65 ± 0.21	2.73 ± 0.41
Myocardial AMP/ADP/ATP (pmols/mg tissue)		
AMP	6.85 ± 6.25	6.44 ± 2.23
ADP	26.86 ± 5.76	27.34 ± 7.19
ATP	18.50 ± 10.03	22.64 ± 7.99
Brain ATP measurements (pmols/mg tissue)		
ATP	0.35 ± 0.06	2.44 ± 1.48*

Note

Values are *M* ± *SDM* and expressed in pmols/µl of whole blood and as pmols/mg of tissue (myocardium and brain). Pyruvate infusion started at 0 min of age (baseline) for 90 min.

Abbreviations: ADP, adenosine diphosphate; AMP, adenosine monophosphate; ATP, adenosine triphosphate.

* *p* < .05 versus Saline group, unpaired *t* test.

## 4 DISCUSSION

Birth asphyxia accounts for substantial mortality and morbidity throughout the world. Limiting neurological and cardiovascular morbidity in the postresuscitation period is currently limited to therapeutic hypothermia, which requires servo‐controlled equipment. A simple intravenous infusion with the potential to reducing morbidity has broad therapeutic potential. Pyruvate is a novel agent in the pharmacotherapy during resuscitation and postresuscitation phases, linking its antioxidant and energy‐restoration properties to its beneficial actions in the metabolically challenged myocardium. We evaluated the cardioprotective and neuroprotective potential of pyruvate in a perinatal model of asphyxial CA. We found that pyruvate administration might improve cerebral metabolic oxygen consumption, in a model of asphyxial CA in newborn lambs.

Pyruvate usually is not an essential myocardial fuel because of submillimolar plasma concentrations, but the heart is responsive functionally and metabolically to supraphysiologic levels of exogenous pyruvate (Mallet et al., 2005). Intravenous pyruvate administration during CA and ROSC temporarily increased GSH/GSSG and phosphocreatine phosphorylation potential, resulting in an electromechanical and metabolic recovery in dogs (Sharma et al., 2005). Pyruvate enhances myocardial performance in isolated or perfused heart preparations (Chen, London, Murphy, & Steenbergen, 1998; Martin, Valdivia, Bunger, Lasley, & Mentzer, 1998). Intracoronary pyruvate improved systolic and diastolic myocardial function and increased ejection fraction without increasing heart rate in adult patients with congestive cardiac failure (Hermann et al., 2004). In contrast, we did not find any difference in systolic and diastolic blood pressures and stroke volume between the two groups. Echocardiography, not performed in our study, could have yielded a better assessment of myocardial function. Biologic variability in response to asphyxial insult may also contribute to the absence of difference between the groups.

In contrast, no distinct differences in tissue lactate between the two groups may explain suboptimal myocardial response in terms of systemic hemodynamics, as the rate of disappearance of lactate may be an indicator of organ injury due to asphyxia (Eriksen, Trautner, Hahn, & Greisen, 2019). No differences in myocardial ATP measurements between the groups suggest that oxidative phosphorylation and hence oxygen consumption is depressed in the asphyxiated myocardium despite pyruvate infusion. The stress of perinatal transition along with an additional burden of perinatal asphyxia may prolong the recovery time of the dynamic myocardium despite interventions such as pyruvate. Future studies looking at later time points may offer insights into the effects of pyruvate on the myocardium.

The effects of pyruvate on the brain were distinct from the one on the heart. Factors such as hypotension and hypocarbia that produce changes in cerebral blood flow (CBF) and oxygen consumption (CMRO_2_) are critical to the evolution of brain injury in perinatal asphyxia (Rosenberg, 1988a, 1988b; Rosenberg, Parks, Murdaugh, & Parker, 1989). In our study, CMRO_2_ was depressed in the first 90 min after asphyxia insult in both the groups. Reversible inhibition of mitochondrial function, in both nonsynaptic and synaptic mitochondria in the immediate postasphyxial period, may explain depressed CMRO_2_ in both the groups (Rosenberg et al., 1989). Severe depression of CMRO_2_ has been reported in neonatal piglets with hypoxic‐ischemic insult with a significant reduction by 4 hr, which sustained over a 24‐hr period (Tichauer et al., 2009). CMRO_2_ values in the pyruvate group are consistent with those reported in the literature in asphyxiated newborn lambs (Rosenberg, 1988a, 1988b), suggesting improvements in oxygen utilization from extremely depressed levels seen postasphyxia. Our study is limited by the absence of control lambs; however, control nonasphyxiated lambs have CMRO_2_ values similar to asphyxiated lambs (Rosenberg, 1988b), both reportedly higher than that reported in the pyruvate group. Unpublished studies from our laboratory also suggest nonasphyxiated lambs have CMRO_2_ (0.84 ± 0.11 ml kg^−1^ min^−1^) similar to asphyxiated group‐administered pyruvate (0.9 ± 0.08 ml kg^−1^ min^−1^). Also, data from our laboratory suggest that fetal CMRO_2_ before asphyxiation are similar (0.43 ± 0.09 ml kg^−1^ min^−1^) between the two asphyxiated groups. We also report similar FiO_2_ values (0.21–0.4) in the postasphyxial period in both groups. We can speculate from both the present and previous studies that an increase in (and restoration of) oxygen consumption in the brain from extremely depressed levels is likely resulting from efficient oxygen utilization in the brain, resulting in higher ATP generation in the pyruvate group. Nonetheless, the potential for the increase in CMRO_2_, leading to higher oxidative stress, needs further exploration.

**FIGURE 5:**
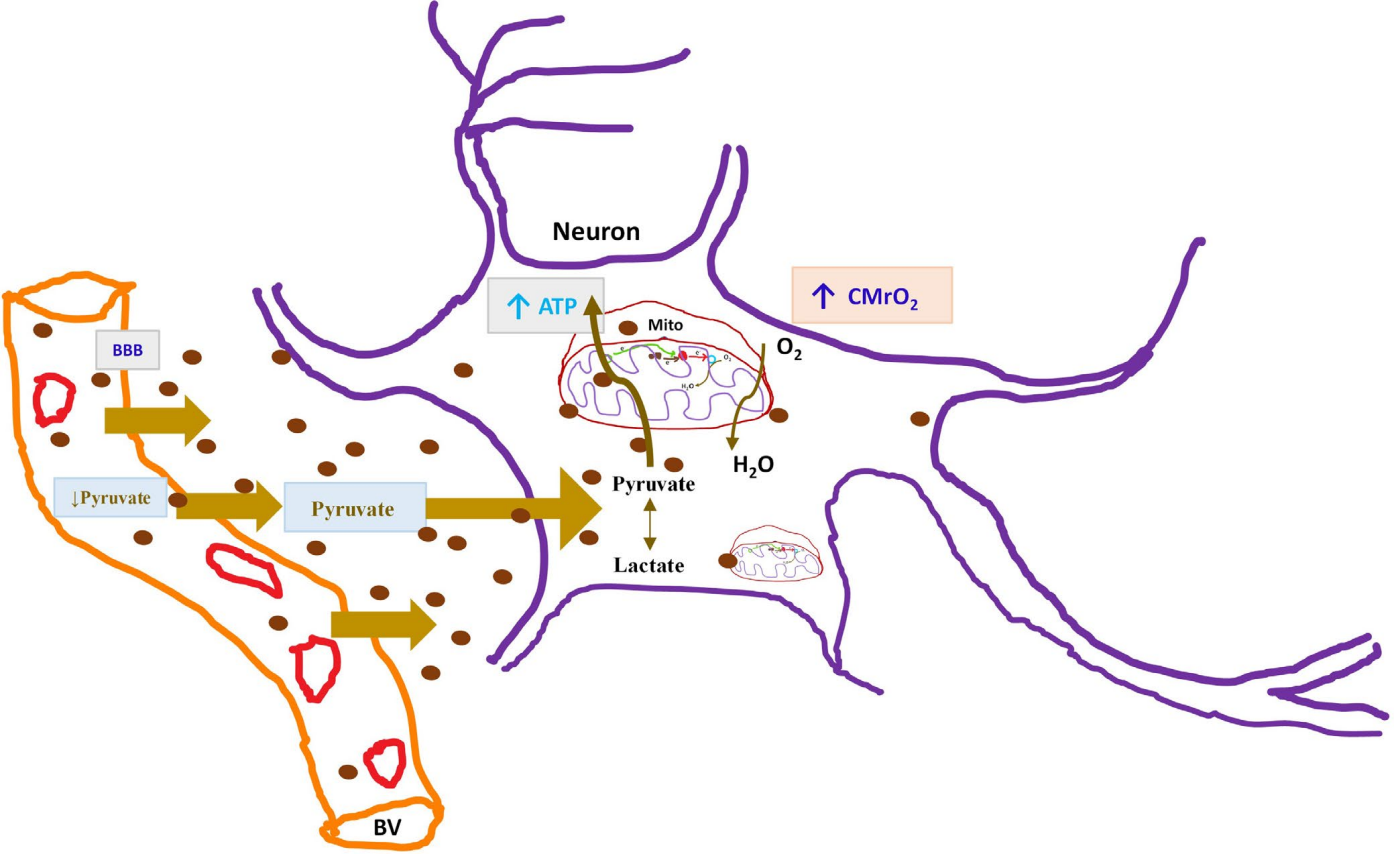
Illustration of pyruvate's mechanism of action. The rapid uptake of pyruvate by the neurons through the blood–brain barrier (BBB) leading to higher cerebral metabolic oxygen consumption (CMRO_2_), resulting in adenosine triphosphate (ATP) generation by the mitochondrial aerobic respiration via the Krebs's cycle. BV, blood vessel

Pyruvate, the product of glycolysis, enters the Krebs cycle as acetyl CoA and, in the presence of oxygen, is utilized almost entirely for the oxidation of carbohydrates to generate ATP. Pyruvate levels in the plasma were lower in the pyruvate group compared to the control group, possibly suggesting rapid uptake on pyruvate into the oxidative phosphorylation cycle, thereby increasing CMRO_2_. The blood–brain barrier transports pyruvate typically at a rate much slower than glucose; however, significant pyruvate entry to the brain is achieved by elevating plasma pyruvate concentrations (Lee, Kim, & Koh, 2001). Pyruvate increases endogenous respiration by suppressing superoxide production by submitochondrial particles and prevents the collapse of mitochondrial membrane potential (Wang et al., 2007). Higher GSH levels from pyruvate also stabilize the inner mitochondrial membrane (Mallet et al., 2005). An intact mitochondrial potential drives the uptake of acetyl Co‐A generated from pyruvate to enter the Krebs cycle resulting in the generation of ATP. Paradoxically, an increase in pyruvate seen in the control group may reflect mitochondrial dysfunction from perinatal asphyxia. Neurons consume about 80% of the ATP, whereas glia‐based processes spend the rest of the energy generated by oxidative phosphorylation (Harris, Jolivet, & Attwell, 2012; Hyder et al., 2006). Energy utilization by the neurons is related to the ionic pumps that reestablish the electrochemical gradients dissipated by signaling, namely, action potentials and synaptic potentials. Recent studies suggest that the majority of the energy used by neurons for signaling appears to be consumed at the synapse (Harris et al., 2012). Pyruvate protects synaptic function against the deleterious effects of hypoglycemia by enhancing glycogen content in astrocytes (Shetty, Sadgrove, Galeffi, & Turner, 2012). Pyruvate, by inhibiting Poly‐ADP ribose polymerase 1, protects the brain from cerebral ischemia and severe hypoglycemia (Suh et al., 2003). The low ATP levels following CA leads to a failure of many of the mechanisms that maintain cell integrity, particularly the sodium/potassium (NA/K) pumps and the machinery that maintains low intracellular calcium (Magistretti, 2009; Magistretti & Pellerin, 1999). When the Na/K pumps fail, an excessive influx of positively charged sodium ions precipitates massive depolarization of neurons that characterize primary energy failure in hypoxic‐ischemic encephalopathy. Pyruvate by increasing ATP generation, as illustrated in our study (Figure 5), may target the pathologies of brain hypometabolism, oxidative stress, and inflammation leading to its neuroprotective effects (Zilberter, Gubkina, & Ivanov, 2015).

The study is limited by the lack of cellular specificity of pyruvate and energy metabolism in the brain, as different cell types have distinctive metabolic profiles (Magistretti & Allaman, 2015). Aerobic glycolysis and lactate production are metabolic features of astrocytes and marginally expressed in neurons (Magistretti & Allaman, 2015). Astrocytes containing glycogen are highest in regions of maximal synaptic density, such as the gray matter, medullar, hippocampus, midbrain, thalamus, and striatum (Falkowska et al., 2015). We did not measure ATP in specific regions of the brain, which can vary depending on the severity and the nature of HI to the brain following CA. Pyruvate infusion to achieve supraphysiologic plasma concentrations may have provided the necessary substrate for oxidative phosphorylation to occur, generating ATP. The doses of pyruvate administered are in line with that previously reported in the literature (Kristo et al., 2004; Mongan et al., 2002). Administration of high doses of SP did not lead to either hypernatremia or hyperlactatemia, as both the groups had similar concentrations of sodium and lactate. Additional markers of energy failure, such as functional MRI or MRS activation patterns, were not performed to confirm the findings. Monitoring of lambs for a short period limited our conclusions, especially on the myocardium; more extended periods of monitoring might have revealed additional differences between the groups. We estimated cardiac output using pulmonary blood flow as the instrumentation of ascending aorta is technically challenging in neonatal lambs. The absence of cardioprotective effects may suggest that the pyruvate responses on the brain may be independent of the myocardium in the postresuscitation period. Despite these limitations, we have shown for the first time that pyruvate administration may have beneficial effects on brain metabolism in a newborn model of asphyxial CA with profound respiratory and metabolic acidosis.

We conclude that in an experimental model of asphyxial CA in newborn lambs, pyruvate infusion increased CMRO_2_ in the brain with a resultant increase in brain energy stores. The rapid uptake of pyruvate by the neurons not only provides substrate critical for oxidative phosphorylation but also might stabilize the mitochondrial membrane attenuating mitochondrial dysfunction. Resulting higher energy stores are essential to protect synaptic function and to alleviate neuronal dysfunction and neuronal loss. The study opens up new avenues for decreasing morbidity and mortality associated with CPR in neonates and children and warrants further clinical trials.

## CONFLICT OF INTEREST

The authors declare that they have no competing interests.

## AUTHOR CONTRIBUTIONS

Experiments designed by V.H.K and S.L. Experimental protocol and hemodynamic measurements performed by S.G., C.K., J.H., V.H.K., and P.C. Pyruvate preparation & ATP assay by L.N. Analysis and interpretation of data by S.G., C.K., L.N., J.H., P.C., V.H.K., and S.L. Manuscript revised critically for intellectual content by S.G., C.K., L.N., J.H., P.C., V.H.K., and S.L. All authors read and approved the final version of the manuscript.

## ETHICS APPROVAL AND CONSENT TO PARTICIPATE

The study was approved by the Institutional Animal Care and Use Committee of the University at Buffalo, Buffalo, NY (IACUC# PED10085N).

## CONSENT FOR PUBLICATION

Not applicable (animal study).

## Data Availability

The datasets used and analyzed during the current study are available from the corresponding author on reasonable request.
